# Making the most of Artificial Intelligence and Large Language Models to support collection development in health sciences libraries

**DOI:** 10.5195/jmla.2025.2079

**Published:** 2025-01-14

**Authors:** Ivan Portillo, David Carson

**Affiliations:** 1 iportillo@chapman.edu, Health Sciences Librarian, Director of Rinker Campus Library Services, Leatherby Libraries, Chapman University, Irvine, CA; 2 carsondav@ohsu.edu, Health Sciences Education & Research Librarian, Oregon Health & Science University, Portland, OR

**Keywords:** Generative artificial intelligence, large language models, ChatGPT, Microsoft Copilot, Perplexity, Google Gemini, collection development, collection assessment, health sciences libraries

## Abstract

This project investigated the potential of generative AI models in aiding health sciences librarians with collection development. Researchers at Chapman University's Harry and Diane Rinker Health Science campus evaluated four generative AI models—ChatGPT 4.0, Google Gemini, Perplexity, and Microsoft Copilot—over six months starting in March 2024. Two prompts were used: one to generate recent eBook titles in specific health sciences fields and another to identify subject gaps in the existing collection. The first prompt revealed inconsistencies across models, with Copilot and Perplexity providing sources but also inaccuracies. The second prompt yielded more useful results, with all models offering helpful analysis and accurate Library of Congress call numbers. The findings suggest that Large Language Models (LLMs) are not yet reliable as primary tools for collection development due to inaccuracies and hallucinations. However, they can serve as supplementary tools for analyzing subject coverage and identifying gaps in health sciences collections.

Artificial intelligence (AI) and large language models (LLMs) have garnered significant interest since the public launch of ChatGPT in 2022 [1]. LLMs and generative AI models have made substantial strides in their capabilities, now offering references and detailed analyses of uploaded files in their responses. These advancements present a promising opportunity for librarians to potentially reduce workload and increase efficiency [2, 3]. This project was designed to explore the potential of generative AI models in assisting health sciences librarians with collection development, particularly in identifying gaps and recommending book titles.

Chapman University is a private university with two campuses in Orange County, California, with approximately 10,000 students and 2,000 staff and faculty. The researchers are health sciences librarians based at the Harry and Diane Rinker Health Science campus in Irvine, CA, which serves primarily graduate and doctoral students in physical therapy, physician assistant, communication sciences, and pharmacy programs. Beginning in March 2024, the researchers evaluated four generative AI models over a period of six months using two prompts designed by the researchers to aid librarians in collection development. The four generative AI models assessed included ChatGPT 4.0, Google Gemini, Perplexity, and Microsoft Copilot.

The first prompt used in each generative AI model sought to generate a list of recent eBook titles published in the last two years focused on physical therapy, physician assistant, communication sciences and disorders, and pharmacy. The second prompt sought to identify subject gaps in an existing collection and create a list of recommended call number ranges. To accomplish this task, a list of the library's collection was uploaded into each generative AI model. The list was created using the Create List function within Sierra, an Integrated Library System from Innovative. The list was exported as a CSV file with fields for title, Library of Congress call number, location, and item status. If the AI model did not accept CSV files, such as the non-premium versions of Perplexity and Google Gemini, the researchers copied and pasted the list of titles and Library of Congress call numbers from the collection into the prompt field.

The results were assessed based on quality, accuracy, the presence of fabricated titles (often referred to as “hallucinations”), if references were provided, correct citation details, and accurate Library of Congress (LC) call numbers. Each AI model produced inconsistent results for the first prompt. Five titles per subject were generated by each AI model, with Copilot and Perplexity being the only two that provided sources for the titles generated. Perplexity generated inaccurate details, including publication years, DOIs, and publishers. Copilot was the most accurate, while Gemini and ChatGPT provided inaccuracies and hallucinations. It should be noted that the researchers found that all four AI models generated hallucinations and inaccurate information on previous dates with the same prompt provided. While Perplexity and Copilot performed the best, the researchers would not recommend any generative AI models for title recommendations due to inaccuracies and inconsistencies.

The researchers found the second prompt more helpful from each of the four generative AI models. Each AI model provided helpful analysis and accurate LC call numbers. Each AI model provided minor differences in the subject gaps they identified, but all provided the reasoning behind the importance of each subject area recommended. For example, when asked to identify subject gaps for physical therapy, ChatGPT and Copilot agreed on eight broad subject gaps, such as kinesiology and geriatrics. Perplexity and Gemini offered narrower, more specific suggestions, such as telehealth and electrophysical agents. The researchers have found this to be useful in their current collection development cycle.

Overall, the results reinforce the notion that LLMs are not yet suitable as primary information retrieval systems in collection development. It should be noted that the researchers found that all four LLMs generated hallucinations for prompt #1 and inaccurate information on previous dates. Responses also varied for prompt #2 depending on the day or time queried. The researchers still found that generative AI models can serve as a supplementary tool for analyzing the subject coverage of their collection, identifying subject gaps, and highlighting areas for health science programs that may not be as well represented in a library collection.
